# Testing the potential significance of different scion/rootstock genotype combinations on the ecology of old cultivated olive trees in the southeast Mediterranean area

**DOI:** 10.1186/s12898-017-0114-3

**Published:** 2017-02-06

**Authors:** Oz Barazani, Yoni Waitz, Yizhar Tugendhaft, Michael Dorman, Arnon Dag, Mohammed Hamidat, Thameen Hijawi, Zohar Kerem, Erik Westberg, Joachim W. Kadereit

**Affiliations:** 1 Institute of Plant Sciences, Israel Plant Gene Bank, Agricultural Research Organization, 75359 Rishon LeZion, Israel; 2 Department of Fruit Tree Sciences, Institute of Plant Sciences, Agricultural Research Organization, Gilat Research Center, 85280 M.P. Negev 2, Israel; 3 Institute of Biochemistry, Food Science and Nutrition, Faculty of Agricultural, Food and Environmental Quality Sciences, The Hebrew University of Jerusalem, 76100 Rehovot, Israel; 4 Department of Geography and Environmental Development, Ben-Gurion University of the Negev, 84105 Beer-Sheva, Israel; 5 Arab Agronomist Association, Al Nahda St., Ramallah and Al-Bireh Governorate, 4504 Al-Bireh, Palestine; 6 Institut für Spezielle Botanik und Botanischer Garten, Johannes Gutenberg-Universität Mainz, 55099 Mainz, Germany

**Keywords:** Akaike information criterion (AIC) selection model, Environmental conditions, Multi-locus lineage analysis, Olive oil quality, Selection

## Abstract

**Background:**

A previous multi-locus lineage (MLL) analysis of SSR-microsatellite data of old olive trees in the southeast Mediterranean area had shown the predominance of the Souri cultivar (MLL1) among grafted trees. The MLL analysis had also identified an MLL (MLL7) that was more common among rootstocks than other MLLs. We here present a comparison of the MLL combinations MLL1 (scion)/MLL7 (rootstock) and MLL1/MLL1 in order to investigate the possible influence of rootstock on scion phenotype.

**Results:**

A linear regression analysis demonstrated that the abundance of MLL1/MLL7 trees decreases and of MLL1/MLL1 trees increases along a gradient of increasing aridity. Hypothesizing that grafting on MLL7 provides an advantage under certain conditions, Akaike information criterion (AIC) model selection procedure was used to assess the influence of different environmental conditions on phenotypic characteristics of the fruits and oil of the two MLL combinations. The most parsimonious models indicated differential influences of environmental conditions on parameters of olive oil quality in trees belonging to the MLL1/MLL7 and MLL1/MLL1 combinations, but a similar influence on fruit characteristics and oil content. These results suggest that in certain environments grafting of the local Souri cultivar on MLL7 rootstocks and the MLL1/MLL1 combination result in improved oil quality. The decreasing number of MLL1/MLL7 trees along an aridity gradient suggests that use of this genotype combination in arid sites was not favoured because of sensitivity of MLL7 to drought.

**Conclusions:**

Our results thus suggest that MLL1/MLL7 and MLL1/MLL1 combinations were selected by growers in traditional rain-fed cultivation under Mediterranean climate conditions in the southeast Mediterranean area.

**Electronic supplementary material:**

The online version of this article (doi:10.1186/s12898-017-0114-3) contains supplementary material, which is available to authorized users.

## Background

The history of fruit tree domestication is strongly linked to grafting, which provided an easy technique for clonal reproduction of trees with desirable properties that are difficult to propagate vegetatively. It is generally accepted that the domestication of several fruit trees such as apple, plum and others was not possible without the development of the grafting technique [1]. In addition to being useful or necessary for propagation, rootstocks can influence the size of scions and increase their vigor. Physiological investigation of the interactions between scions and rootstocks in grapevine showed that grafting can reduce the toxic effects of salinity by the ability of the rootstock to limit the uptake of Na^+^ and Cl^−^ ions by the scion [2]. In several crop species, e.g., in peach [3] and grapevine [4], rootstocks have been shown to reduce leaf chlorosis caused by iron deficiency. Rootstocks also have been selected to increase tree tolerance to abiotic stresses such as drought and soil pH [1], and increase the resistance against soil pathogens [5].

Genetic comparison of suckers and the canopy of old olive trees in the Iberian peninsula [6] and the southeast Mediterranean area [7] provide strong evidence for grafting as a common practice in olive cultivation in the past [8, 9]. Diez et al. suggested that by grafting of scions on wild growing trees, natural populations of *Olea europaea* subsp. *europaea* var. *sylvestris* were transformed into olive groves [6], and the use of individual ‘wild’ olive trees as vigorous rootstocks in traditional olive cultivation has also been suggested [9]. However, evidence on the potential contribution of the rootstock to olive tree fitness and phenotypic properties is very limited and is based on recent experimental systems using combinations of known cultivars [10–13]. In these experiments, particular combinations of rootstocks and scions were shown to decrease the harmful effects of excessive boron concentrations in the soil [11], and to increase resistance to *Verticillium* wilt [10, 12].

Previously we reported that most old olive trees in the southeast Mediterranean area are grafted. In addition, a multi-locus lineage (MLL) analysis had shown that most of the scions (ca. 90%) belong to a single MLL (MLL1), presumably representing the Souri cultivar, and that most of the rootstocks probably originated from plant individuals resulting from sexual reproduction [7]. However, we also identified an MLL (MLL7) that was more common than other MLLs in rootstocks of grafted old olive trees and was present in 23% of the trees analysed [7]. This led to the hypothesis that olive cultivation in the region may have involved selection not only of a specific scion but also of a specific rootstock. Traditional olive groves in the southeast Mediterranean area are distributed along a geographic gradient of diverse climatic, topographic and edaphic conditions [14, 15]. As rootstocks might have been selected for improvement of the root system in stressful conditions and/or for their influence on phenotypic properties of the scion, we aimed to investigate the contribution of the most common rootstock (MLL7) to the fitness and phenotype of olive trees in different environments. We here use a model selection procedure based on the Akaike information criterion (AIC) to investigate the potential advantage of the MLL1/MLL7 combination by quantifying the impact of a number of environmental variables on several agriculturally important phenotypic traits.

## Methods

In our previous study [7] we reported on a total of 249 old olive trees in which both suckers and scions were collected from the same trees and genotyped. Identification of scion and rootstock MLLs was performed with leaf samples taken from tree canopies (i.e. scions) and from suckers that developed from the trunk base [7]. Thus, a comparison between scion and sucker of the same tree enabled us to differentiate between three genetic groups (GG): 1) GG1 included trees in which the common Souri cultivar (MLL1) was grafted on MLL7 (49 trees); 2) GG2, in which both suckers and scions were assigned to MLL1 (62 trees); and 3) GG3 included those trees in which the common Souri cultivar was grafted on single-occurrence rootstock MLLs that probably originated from sexual reproduction (117 trees). Trees of the second group (GG2) were either the result of vegetative propagation of MLL1 or of grafting of MLL1 scions on MLL1 truncheons. The analysis included a total of 228 old olive trees from 31 groves with various environmental conditions in the southeast Mediterranean area.

### Phenotypic characterization

Fruits were collected during the harvest in a single season in 2008 and were used for morphological evaluation, oil extraction and evaluation of content and quality of the oil. Morphological evaluation included the weight and dimension of 10 fruits and stones of each tree. The Abencor system (MC2 Ingenieria Y Sistemas, Spain) was used to extract oil from 1 kg of fruits of each of the investigated trees [16], and the relative content of oil and paste water was determined after Soxhlet chemical extraction as previously described [17]. Fatty acid (FFA) profiles were determined following [18], and the ratio between monounsaturated fatty acids (MUFA) and polyunsaturated fatty acids (PUFA) was determined. Following Ben-Gal et al. [18], the peroxide value (milliequivalents of active oxygen per kilogram oil; mequiv kg^−1^), acidity (% free oleic acid) and total content of polyphenols (mg kg^−1^ oil) in the oil were also determined.

### Environmental parameters

Average annual rainfall and elevation data were gathered from the Geographic Information System center database (Hebrew University of Jerusalem) using the lati-/longitudinal coordinates of groves. Daily temperatures for 2008 were collected using the MODIS remote sensing of surface and canopy temperatures (http://modis-land.gsfc.nasa.gov/); *T*_max_ and *T*_min_ were calculated according to Blum et al. [19] and used to determine the number of growing degree days (GDD) during the period from first flowering to fruit harvest (mid-April to mid-November), following the equation $\frac{{T_{{\rm max} } + T_{{\rm min} } }}{2} - T_{base}$ (9.1 °C) [20]). Calcium carbonate content in the soil was used to assess edaphic conditions, as calcareous soil is one of the limiting factors of agricultural practice in the region [15]. Soil samples, five in each grove, were collected from three soil depths (0–30, 30–60 and 60–90 cm), and soil analysis was conducted by the Gilat Extension Services Laboratory and Research Center (Israel); the results are presented as the average value of the three layers analysed (% of CaCO_3_).

### Statistical analysis

Linear regression was used to examine the abundance of trees belonging to the two scion/sucker combinations MLL1/MLL7 (GG1) vs. MLL1/MLL1 (GG2) along gradients of environmental conditions. Linear models were also used to examine which environmental factors explain phenotypic variation. To evaluate the environmental effects, we used a model selection procedure based on the Akaike information criterion (AIC), corrected for small sample sizes [21]. Models where the given trait (e.g. oil content, peroxide value, etc.) was explained using all possible combinations of environmental factors (e.g. GDD, CaCO_3_, etc.), genetic groups (GG1 vs. GG2) and the interactions of each environmental factor with genetic groups were evaluated based on AIC. Old olive trees belonging to GG3 were not included in the AIC analysis as they do not represent a genetically homogenous group. The AIC analysis thus included a total of 111 trees. Factors present in the most parsimonious model (i.e. with the lowest AIC) and their direction of influence were then summarized. The inclusion of interaction terms enabled us to understand whether the genetic groups (GG1 in comparison to GG2) differ in their response to the different environmental factors (i.e. presence of a genotype-by-environment interaction). All statistical analyses were done using R [22].

## Results

### The abundance of olive trees with different genotype combinations in groves along environmental gradients

**Fig. 1:**
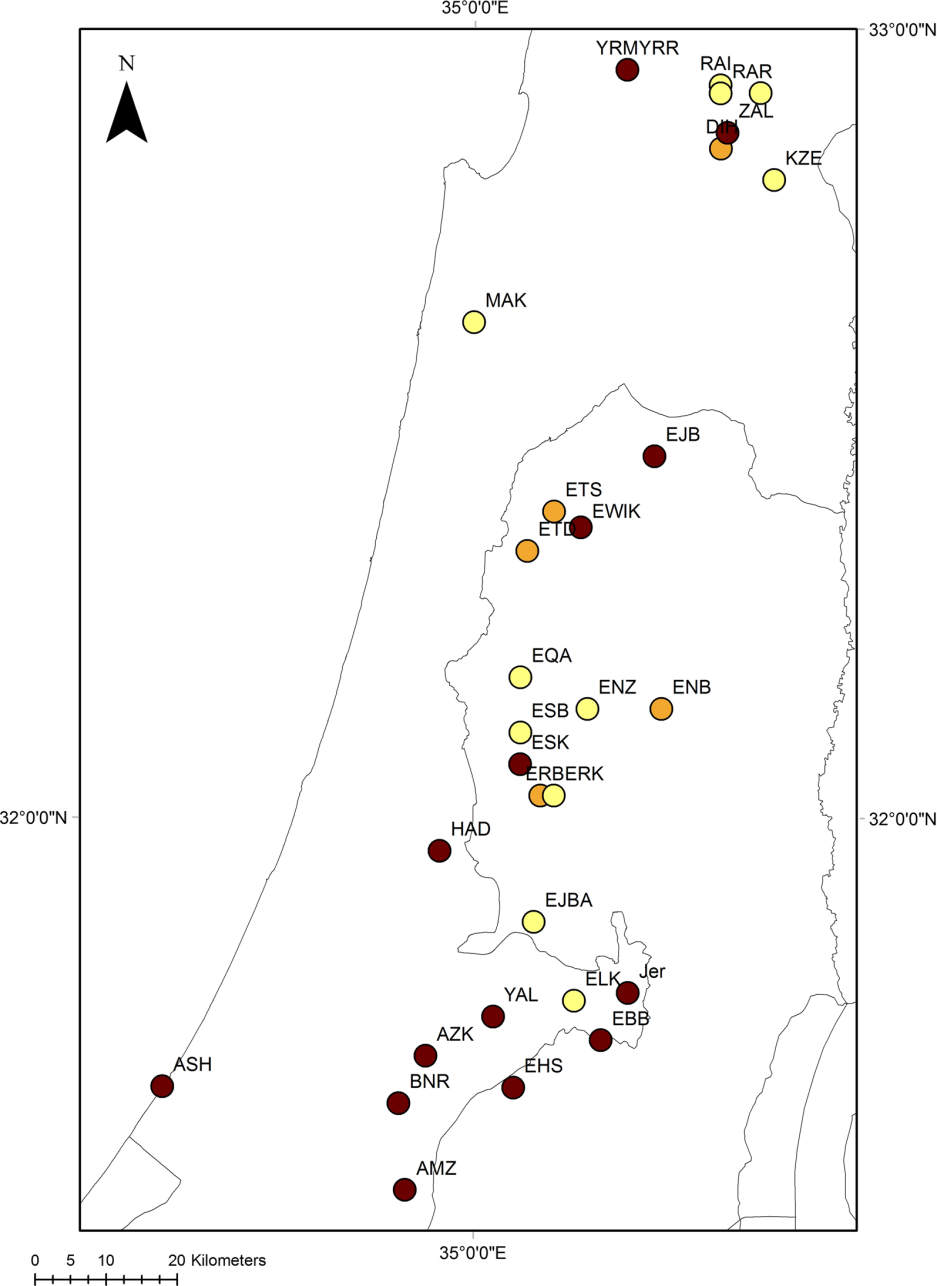
Map showing the sampled groves (c.f. Table 1) and the ratio between trees of the MLL1/MLL7 and MLL1/MLL1 scion/sucker combinations (GG1:GG2) at each site; the *different colours* illustrate the ratio between the two groups, ranging from *yellow* (dominance of GG1 trees) to *brown* (dominance of GG2 trees)

Traditional rain-fed olive groves in the southeast Mediterranean area are scattered through geographical districts that vary in climatic conditions and soil texture and chemistry. For this study, olive groves were selected to represent an aridity gradient from north to south, ranging from relatively mesic Mediterranean climate sites (≥450 mm rainfall) to semi-arid (350–450 mm) and arid conditions with less than <350 mm rainfall per year (ASH) (Table 1, Fig. 1). We included groves of relatively high elevation in the Samaria and Judean Mts. (440 to ca. 720 m a.s.l.) to lower elevation in the inner plain (110–290 m a.s.l.), the Carmel and the coastal plain (MAK, 84 m a.s.l. and ASH 23 m a.s.l., respectively). Variation in the average annual maximum and minimum temperatures (expressed as growing-degree day, GDD) was also found among sites. The range of CaCO_3_ content in the soil (2.0–57.7%) represents the variability of edaphic conditions in the region (Table 1). Linear regression did not show any significant correlation between any of the environmental parameters (data not shown).

**Table 1:** Environmental data of the investigated groves arranged along an aridity gradient; GDD data for ASH is missing

	Geographic region	CaCO_3_ (%)	Soil type	GDD	Average annual rainfall (mm)	Elevation (m a.s.l.)
YRM	Galilee	25.67	Terra Rosa	3365	324.90	341.67
YRR	Galilee	25.67	Terra Roas	3365	324.90	341.67
RAI	Galilee	2.00	Terra Rosa	3655	391.60	379.92
RAR	Galilee	2.67	Terra Rosa	3736	391.60	347.13
ZAL	Galilee	4.33	Terra Rosa	4002	391.60	92.69
DIH	Galilee	34.00	Terra Rosa	3769	391.60	385.75
KAM	Galilee	18.67	Terra Rosa	3631	391.60	329.75
KZE	Galilee	8.00	Heavy soil	4014	391.60	111.38
MAK	Carmel	14.00	Terra Rosa	3425	388.90	84.27
EJB	Samaria	57.67	Terra Rosa	2861	317.50	248.49
ETS	Samaria	28.33	Rendzina	3745	454.50	242.04
EWIK	Samaria	37.00	Terra Rosa	3738	454.50	339.05
ETD	Samaria	45.33	Terra Rosa	3786	454.50	337.72
EQA	Samaria	55.33	Terra Rosa	3573	408.10	286.00
ENZ	Samaria	48.67	Terra Rosa	3446	356.10	373.01
ENB	Samaria	37.67	Heavy soil	2481	356.10	522.56
ESB	Samaria	38.67	Terra Rosa	3372	408.10	289.72
ESK	Samaria	41.00	Terra Rosa	3124	408.10	337.72
ERK	Samaria	38.00	Rendzina	2709	466.10	460.75
HAD	Inner plain	35.33	Rendzina	3821	276.00	111.17
ERB	Judean Mts	54.33	Rendzina	3334	466.10	439.33
EJBA	Judean Mts.	33.67	Terra Rosa	2650	382.90	575.53
JER	Judean Mts.	46.00	Terra Rosa	4077	353.40	723.71
EIK	Judean Mts.	15.33	Terra Rosa	3297	382.90	630.54
YAL	Judean Mts.	38.67	Terra Rosa	3350	287.10	615.27
EBB	Judean Mts.	33.00	Terra Rosa	2133	359.70	726.96
AZK	Inner plain	39.33	Rendzina	3650	320.40	272.65
BNR	Inner plain	24.33	Rendzina	3714	320.40	292.64
EHS	Judean Mts.	25.33	Terra Rosa	2898	301.40	547.66
ASH	Coastal plain	34.33	Sand	–	193.90	23.65
AMZ	Inner plain	42.00	Rendzina	3608	344.50	328.08

**Fig. 2:**
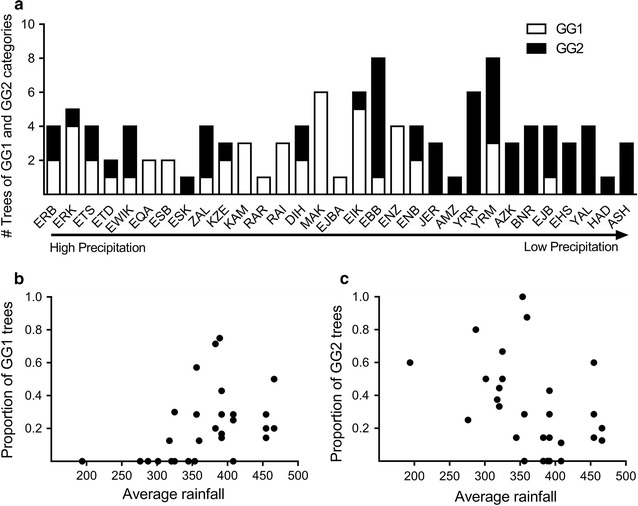
Relative proportions of trees belonging to GG1 and GG2 along an aridity gradient showing the predominance of trees of GG1 in groves in geographic locations with relatively higher average annual rainfall and their absence from the driest groves (**a**). Relation between average annual rainfall and the proportion of trees of GG1 (**b**) and GG2 (**c**); the relative number of trees was calculated from the total number of trees at each site (i.e. including all three categories: GG1, GG2 and GG3)

Mapping the relative number of trees of all three MLL combinations in the investigated groves showed that trees of the MLL1/MLL7 combination (GG1) are more abundant in the northern (Galilee) and central parts (Samaria and Judean Mts.) of the cultivation area of olives in the southeast Mediterranean area (Fig. 1). The linear regression showed a significant association between average rainfall and the proportion of trees belonging to GG1 (*F*_*1,29*_ = 7.068, *P* = 0.0126) and those of GG2 (*F*_*1,29*_ = 6.929, *P* = 0.0135), but this proportion was not associated with any of the other environmental parameters considered, i.e. GDD, elevation (m a.s.l) and the relative content of CaCO_3_ in the soil (Table 2). As expected when estimating the effect of single environmental factor in ecological studies, the significant association between average rainfall and the proportion of GG1 and GG2 trees (*R* ^2^ = 0.19) suggests that other unknown environmental factor(s) contribute to the abundance of these two MLL combinations along the aridity gradient. Nevertheless, the abundance of trees of GG1 was higher at more mesic locations, while the proportion of trees belonging to GG2 increased with increasing aridity (Fig. 2).

**Table 2:** Linear regression between environmental parameters and the relative number of old olive trees belonging to GG1 and GG2

	GG1			GG2		
	*R* ^2^	*F*_*1,29*_	*P*	*R* ^2^	*F*_*1,29*_	*P*
Average rainfall	0.1960	7.0680	0.0126	0.1928	6.9290	0.0135
Elevation	4.26 × 10^−5^	0.0012	0.9722	0.1148	3.7610	0.0623
GDD	0.01120	0.3285	0.5710	0.0061	0.1716	0.6818
Calcium	0.07734	2.4310	0.1298	0.0211	0.6257	0.4353

### Testing the potential contribution of genotype combination to tree phenotype

ANOVA post hoc tests did not show significant differences between trees belonging to the GG1 and GG2 combinations in any of the eight phenotypic traits measured (Additional file 1: Figure S1, Additional file 2: Table S1). When taking into account environmental effects and genetic group in the AIC model selection procedure, the content of paste water in the fruit, the peroxide value and stone length were positively associated with grafting, while the ratio of MUFA/PUFA and stone width were negatively influenced by grafting of MLL1 on MLL7 (Table 3). Note that the use of positive or negative ‘influence’ or ‘effect’ refers to their statistical term in the model, and thus does not necessarily reflect beneficial (positive) or detrimental (negative) effects. Our results further showed that the environmental parameters influenced most of the fruit traits of GG1 and GG2 trees in a similar way (Table 3 and Additional file 3). Exceptions to this were the positive effects of elevation and CaCO_3_ content on paste water and peroxide value in trees of GG1, respectively, and the negative effects of these environmental parameters on these traits in trees of GG2. Oil content and fruit and stone dimension and weight were positively influenced by average rainfall in both GG1 and GG2 trees. Similar negative effects of average rainfall were found in acidity and paste water content, while the average rainfall did not have any effect on the other oil quality characters in the two groups of trees (i.e. total polyphenol concentration, peroxide values and MUFA/PUFA). Elevation had a stronger effect on MUFA/PUFA in trees of the GG1 combination than in those belonging to GG2 and on peroxide values in GG2 trees than in trees of GG1. Similarly, CaCO_3_ had a stronger influence on stone weight in GG1 trees and on stone length in trees belonging to GG2. GDD had a positive effect on peroxide values in both groups of trees, but its effect was more pronounced in trees of GG2. In addition, the negative effect of GDD on stone weight was stronger in trees belonging to GG1 than in those belonging to GG2. The adjusted *R* ^2^ values of the AIC model showed the lowest value for total concentration of polyphenols (0.03) and the highest for peroxide value (0.40) and stone weight (0.55) (Table 3 and Additional file 3).

**Table 3:**
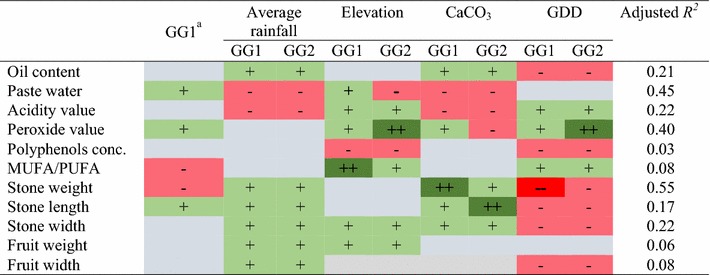
Predicted direction of influence for the effects of environmental variables on the phenotypic traits of the investigated trees belonging to GG1 and GG2; model summaries are provided in Additional file 3

Positive and negative association is given by the coefficients of the most parsimonious model (Additional file 3) for the given trait as either positive (+ green cells), negative (− red cells) or zero (blank grey cells; when the variable was not present in the final model). Interaction between genotype combination and environmental effects is indicated by the differences between the two columns for a given environmental variable. For example, the effect of CaCO_3_ on peroxide value is positive in GG1 but negative in GG2 due to the presence of an interaction term in the final model

^*^Results of general linear model explaining the effect of genotype combination on phenotypic traits

## Discussion

Old olive trees in the southeast Mediterranean area that belong to the common Souri cultivar have rootstocks of different genotype (MLL). Our previous results indicated that 23% of the rootstocks belong to one multi-locus lineage (i.e. MLL7) [7]. Mapping of the trees in which the local MLL1 variety is grafted on the common MLL7 (GG1), and those in which scion and suckers both belong to MLL1 (GG2) showed that the first group of trees (GG1) are more abundant in the northern and central parts of the olive cultivation area in the region (Fig. 1). In addition, the linear regression analysis indicated a significant association between the relative number of GG1 trees at each grove and the average annual precipitation (Fig. 2). Thus, old olive trees growing in groves with different environmental conditions (Table 1), but with the same MLL in their fruit bearing part (MLL1) and with rootstocks belonging to either MLL1 or MLL7, offer an opportunity to examine the possible contribution of the rootstock to traits of the crop, which more commonly is studied in common garden experiments.

Our sampling included trees growing in a range of soils that vary in their CaCO_3_ content (2.0–57.7%) (Table 1). It has been suggested that grafting of olives is recommended for growing trees in problematic soils, such as soils with high CaCO_3_ contents or saline soils [13]. We thus hypothesized that grafting on the specific MLL7 can provide an advantage in the calcareous soils of the region, where soil pH is high and availability of water and micronutrients is limited [15]. Supporting this hypothesis, the AIC model selection procedure showed differential positive and negative responses of peroxide values to the effect of CaCO_3_ in GG1 and GG2 trees, respectively (Table 3). Also, a strong positive influence of elevation on the oleic acid content in the oil, and thus on MUFA/PUFA, of GG1 trees in comparison with GG2 trees was found (Table 3). As peroxides are produced from the oxidation of fatty acids, high levels of peroxide are undesirable, causing rancid taste and reducing the oil shelf-life [23]. Equally, oleic acid (MUFA) is a major determinant of mouth-feel, aroma and shelf life of olive oil [24]. As both taste and shelf life are likely to have been quality criteria important for early farmers, our results, showing differential responses of the two groups of scion/sucker combination in peroxide values and oleic acid contents, suggest a potential contribution of the MLL7 rootstock to olive oil quality under different edaphic conditions.

Results of the AIC-based model selection indicated that all environmental parameters in general had a similar influence on fruit and stone size and weight, and hence on oil content in the fruits, in the two genotype combinations (Table 3). In addition, average rainfall influenced the quality traits (as well as all other phenotypic characters) in GG1 and GG2 trees in a similar way (zero, positive or negative). However, elevation as well as GDD had a stronger positive influence on peroxide values in GG2 than in GG1 trees, and elevation had stronger positive effect on the MUFA/PUFA ratio in GG1 trees as compared to GG2 trees (Table 3). Thus these results provided further evidence, in addition to the effect of elevation and CaCO_3_ described above, that some environmental parameters have a differential influence on oil quality in GG1 vs. GG2 trees. The decreasing abundance of trees belonging to GG1 (i.e. common MLL1 grafted on MLL7) along an aridity gradient (Fig. 2) may imply that grafting on MLL7 may increase sensitivity of olive trees to drought. Support of this hypothesis may be provided by a recent study which showed that young trees that were produced from the common MLL1 showed higher drought tolerance than trees of the Barnea cultivar (no comparison with MLL1/MLL7 was made), suitable for intensive agricultural conditions based on controlled irrigation [25].

## Conclusions

Overall, our results seem to imply that grafting of the common Souri cultivar (MLL1) on the MLL7 rootstock was governed by two opposing forces. On the one hand, growers in the past may have chosen to graft the common cultivar (MLL1) on MLL7 in order to enhance crop performance under certain environments. As MLL7 was used for grafting much more commonly than other MLLs [7], it appears that not grafting alone but grafting on MLL7 has this positive effect, which suggests deliberate rootstock selection. On the other hand, this advantage of grafting on MLL7 is countered by the plausible sensitivity of MLL7 to drought, so that this rootstock was less used in increasingly arid environments. If the scion/rootstock combination MLL1/MLL1 should represent non-grafted trees, it could be concluded that the grafting technique itself was less used in arid environments. Recent studies that assessed oil quality of the Souri cultivar under different irrigation regimes demonstrated the better performance of the cultivar under rain-fed condition [26, 27]. As these oil quality traits reflect the nutritional value of the olive oil, its taste and oxidative status [23, 24], it seems that the scion/rootstock combination MLL1/MLL7 was ideally adapted to conditions in a southeast Mediterranean climate.

## Additional files

**Additional file 1: Figure S1.** Box plot comparisons of phenotypic traits in old olive trees of the MLL1/MLL7 (GG1) and MLL1/MLL1 (GG2) scion/sucker combinations. Traits included the oil content in the fruits, waste water content obtained in the oil extraction process, four oil quality characteristics and three morphological properties of the fruits and stones.

**Additional file 2: Table S1.** Quantitative phenotypic data for each tree belonging to GG1 and GG2; md represent missing data.

**Additional file 3.** Results of the model-selection procedure.

## Abbreviations

AIC: Akaike information criterion
FFA: free fatty acid
GDD: growing degree days
GG: genetic group
MLL: multi-locus lineage
MUFA: monounsaturated fatty acids
PUFA: polyunsaturated fatty acids
SSR: simple sequence repeats
