# Supplementary figures and images for: Testing the potential significance of different scion/rootstock genotype combinations on the ecology of old cultivated olive trees in the southeast Mediterranean area
Source: BMC Ecol. 2017 Feb 6;17:3. doi: 10.1186/s12898-017-0114-3 (PMC5295185; doi:10.1186/s12898-017-0114-3)

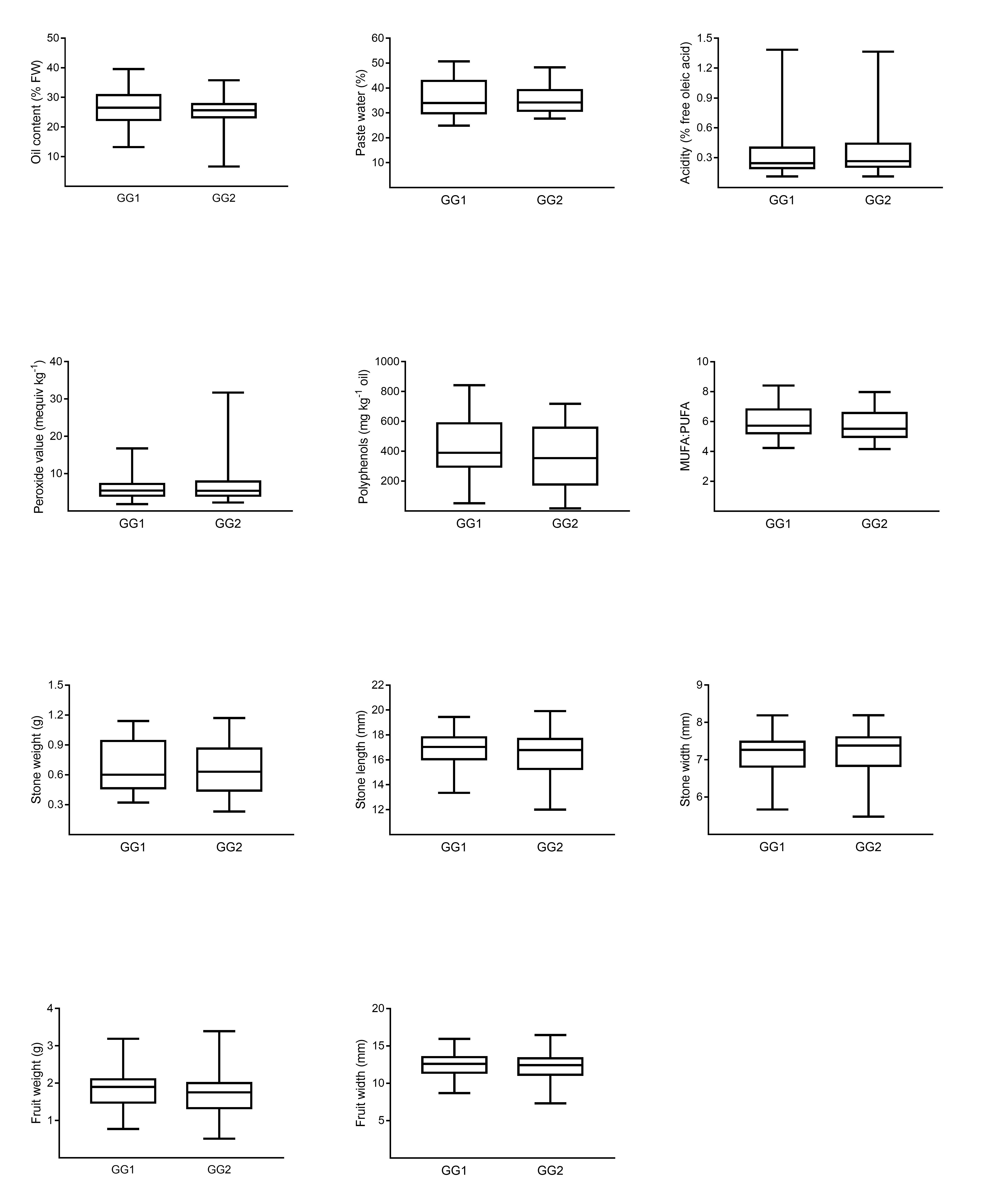

Supplement: Supplementary file 1 — Additional file 1: Figure S1. Box plot comparisons of phenotypic traits in old olive trees of the MLL1/MLL7 (GG1) and MLL1/MLL1 (GG2) scion/sucker combinations. Traits included the oil content in the fruits, waste water content obtained in the oil extraction process, four oil quality characteristics and three morphological properties of the fruits and stones. [file 12898_2017_114_MOESM1_ESM.jpg]
