# Supplementary material for: Testing the potential significance of different scion/rootstock genotype combinations on the ecology of old cultivated olive trees in the southeast Mediterranean area
Source: BMC Ecol. 2017 Feb 6;17:3. doi: 10.1186/s12898-017-0114-3 (PMC5295185; doi:10.1186/s12898-017-0114-3)
Supplement: Supplementary file 3 — Additional file 3. Results of the model-selection procedure. [file 12898_2017_114_MOESM3_ESM.docx]

Appendix

Testing the potential significance of different scion/rootstock genotype combinations on the ecology of old cultivated olive trees in the southeast Mediterranean area

Oz Barazani^1^*, Yoni Waitz^1^, Yizhar Tugendhaft^2,3^, Michael Dorman^4^, Arnon Dag^2^, Mohammed Hamidat^5^, Thameen Hijawi^5^, Zohar Kerem^3^, Erik Westberg^6^ and Joachim W. Kadereit^6^

^1^Institute of Plant Sciences, Israel Plant Gene Bank, Agricultural Research Organization, Bet Dagan 50250, Israel; ^2^Institute of Plant Sciences, Department of Fruit Tree Sciences, Agricultural Research Organization, Gilat Research Center, Israel; ^3^Institute of Biochemistry, Food Science and Nutrition, Faculty of Agricultural, Food and Environmental Quality Sciences, The Hebrew University of Jerusalem, Rehovot 76100, Israel; ^4^Department of Geography and Environmental Development, Ben-Gurion University of the Negev, Beer-Sheva 84105, Israel; ^5^Arab Agronomist Association, Al Nahda St., Ramallah and Al-Bireh Governorate, Al- Bireh 4504, The Palestinian Authority; ^6^Institut für Spezielle Botanik und Botanischer Garten, Johannes Gutenberg-Universität Mainz, D-55099 Mainz, Germany

# Model selection

This document shows the results of a model-selection procedure for the linear model **Y ~ rainfall + elevation + genetic group (GG) + GDD + CaCO_3_ + rainfall:GG + elevation:GG + GDD:GG + CaCO_3_:GG** and all of its sub-models, where **Y** is one of the following dependent variables:

- Oil Content
- Paste water
- Acidity
- Peroxide
- Polyphenols
- MUFA/PUFA
- Stone weight
- Stone length
- Stone width
- Fruit weight
- Fruit width

The model selection procedure involved ranking all models for a given dependent variable based on the Akaike Information Criterion with a correction for finite sample sizes (AICc). The 'best' models for each dependent variable were then summarized.

## Oil Content - Best model

##
## Call:
## lm(formula = oil content ~ CaCO_3_ + GDD + rainfall + 1, data = data)
##
## Residuals:
## Min 1Q Median 3Q Max
## -16.1904 -2.5412 0.0314 2.9303 16.5725
##
## Coefficients:
## Estimate Std. Error t value Pr(>|t|)
## (Intercept) 14.150606 4.846879 2.920 0.00431 **
## CaCO_3_ 0.095211 0.035481 2.683 0.00850 **
## GDD -0.002061 0.001019 -2.022 0.04579 *
## Rainfall 0.041628 0.009916 4.198 5.77e-05 ***
## ---
## Signif. codes: 0 '***' 0.001 '**' 0.01 '*' 0.05 '.' 0.1 ' ' 1
##
## Residual standard error: 5.178 on 102 degrees of freedom
## Multiple R-squared: 0.2303, Adjusted R-squared: 0.2077
## F-statistic: 10.18 on 3 and 102 DF, p-value: 6.384e-06

## Paste water - Best model

##
## Call:
## lm(formula = paste water ~ elevation + CaCO_3_ + GG + rainfall +
## elevation:GG + 1, data = data)
##
## Residuals:
## Min 1Q Median 3Q Max
## -9.5739 -2.5944 -0.1489 2.5135 13.6335
##
## Coefficients:
## Estimate Std. Error t value Pr(>|t|)
## (Intercept) 54.397393 4.102477 13.260 < 2e-16 ***
## Elevation 0.001338 0.003548 0.377 0.70704
## CaCO_3_ -0.233548 0.033213 -7.032 4.38e-10 ***
## GG1 6.174367 2.383396 2.591 0.01123 *
## Rainfall -0.034049 0.010310 -3.303 0.00139 **
## elevation:GG1 -0.013596 0.005612 -2.423 0.01747 *
## ---
## Signif. codes: 0 '***' 0.001 '**' 0.01 '*' 0.05 '.' 0.1 ' ' 1
##
## Residual standard error: 4.659 on 87 degrees of freedom
## Multiple R-squared: 0.479, Adjusted R-squared: 0.449
## F-statistic: 16 on 5 and 87 DF, p-value: 3.735e-11

## Acidity - Best model

##
## Call:
## lm(formula = acidity ~ elevation + CaCO_3_ + GDD + rainfall + 1,
## data = data)
##
## Residuals:
## Min 1Q Median 3Q Max
## -0.35644 -0.12173 -0.03772 0.07024 0.85213
##
## Coefficients:
## Estimate Std. Error t value Pr(>|t|)
## (Intercept) -0.0608993 0.2745011 -0.222 0.824909
## Elevation 0.0003199 0.0001703 1.879 0.063405 .
## CaCO_3_ -0.0043272 0.0015139 -2.858 0.005244 **
## GDD 0.0002264 0.0000591 3.830 0.000231 ***
## Rainfall -0.0008852 0.0004457 -1.986 0.049912 *
## ---
## Signif. codes: 0 '***' 0.001 '**' 0.01 '*' 0.05 '.' 0.1 ' ' 1
##
## Residual standard error: 0.214 on 94 degrees of freedom
## Multiple R-squared: 0.2486, Adjusted R-squared: 0.2166
## F-statistic: 7.774 on 4 and 94 DF, p-value: 1.861e-05

## Peroxide - Best model

##
## Call:
## lm(formula = peroxide ~ elevation + CaCO_3_ + GG + GDD +
## elevation:GG + CaCO_3_:GG + GG:GDD + 1, data = data)
##
## Residuals:
## Min 1Q Median 3Q Max
## -5.5096 -1.8290 -0.5013 0.9681 15.0037
##
## Coefficients:
## Estimate Std. Error t value Pr(>|t|)
## (Intercept) -28.845314 4.979968 -5.792 1.17e-07 ***
## Elevation 0.021536 0.003699 5.821 1.04e-07 ***
## CaCO_3_ 0.151901 0.041189 3.688 0.000400 ***
## GG1 30.419247 7.965941 3.819 0.000256 ***
## GDD 0.006925 0.001154 6.002 4.77e-08 ***
## Elevation:GG1 -0.013388 0.005207 -2.571 0.011900 *
## CaCO3:GG1 -0.173616 0.051762 -3.354 0.001197 **
## GG1:GDD -0.006289 0.001938 -3.245 0.001686 **
## ---
## Signif. codes: 0 '***' 0.001 '**' 0.01 '*' 0.05 '.' 0.1 ' ' 1
##
## Residual standard error: 3.46 on 84 degrees of freedom
## Multiple R-squared: 0.448, Adjusted R-squared: 0.402
## F-statistic: 9.739 on 7 and 84 DF, p-value: 7.971e-09

## Polyphenols - Best model

##
## Call:
## lm(formula = polyphenols ~ elevation + GDD + 1, data = data)
##
## Residuals:
## Min 1Q Median 3Q Max
## -328.26 -161.89 -15.13 144.47 435.08
##
## Coefficients:
## Estimate Std. Error t value Pr(>|t|)
## (Intercept) 882.49938 203.95766 4.327 3.62e-05 ***
## Elevation -0.26724 0.13978 -1.912 0.0588 .
## GDD -0.11227 0.04983 -2.253 0.0265 *
## ---
## Signif. codes: 0 '***' 0.001 '**' 0.01 '*' 0.05 '.' 0.1 ' ' 1
##
## Residual standard error: 201.9 on 99 degrees of freedom
## Multiple R-squared: 0.05263, Adjusted R-squared: 0.03349
## F-statistic: 2.75 on 2 and 99 DF, p-value: 0.06883

## MUFA/PUFA - Best model

##
## Call:
## lm(formula = MUFA/PUFA ~ elevation + GG + GDD + elevation:GG +
## 1, data = data)
##
## Residuals:
## Min 1Q Median 3Q Max
## -1.7506 -0.5816 -0.1049 0.6914 2.1812
##
## Coefficients:
## Estimate Std. Error t value Pr(>|t|)
## (Intercept) 4.2897556 1.0913783 3.931 0.000173 ***
## Elevation 0.0001410 0.0008728 0.162 0.872019
## GG1 -0.7838525 0.4737059 -1.655 0.101713
## GDD 0.0004311 0.0002615 1.649 0.102966
## Elevation:GG1 0.0027291 0.0011479 2.378 0.019700 *
## ---
## Signif. codes: 0 '***' 0.001 '**' 0.01 '*' 0.05 '.' 0.1 ' ' 1
##
## Residual standard error: 0.9661 on 84 degrees of freedom
## Multiple R-squared: 0.122, Adjusted R-squared: 0.08014
## F-statistic: 2.917 on 4 and 84 DF, p-value: 0.02598

## Stone weight - Best model

##
## Call:
## lm(formula = stone wieght ~ CaCO_3_ + GG + GDD + rainfall + CaCO_3_:GG +
## GG:GDD + 1, data = data)
##
## Residuals:
## Min 1Q Median 3Q Max
## -0.3807 -0.1095 -0.0177 0.0947 0.5485
##
## Coefficients:
## Estimate Std. Error t value Pr(>|t|)
## (Intercept) 4.986e-01 1.779e-01 2.802 0.00609 **
## CaCO_3_ 3.454e-03 1.844e-03 1.873 0.06395 .
## GG1 -5.627e-01 2.572e-01 -2.188 0.03101 *
## GDD -2.336e-04 3.810e-05 -6.130 1.72e-08 ***
## Rainfall 2.299e-03 3.424e-04 6.715 1.12e-09 ***
## CaCO_3_:GG1 4.893e-03 2.352e-03 2.080 0.04003 *
## GG1:GDD 1.157e-04 7.003e-05 1.653 0.10153
## ---
## Signif. codes: 0 '***' 0.001 '**' 0.01 '*' 0.05 '.' 0.1 ' ' 1
##
## Residual standard error: 0.1658 on 101 degrees of freedom
## Multiple R-squared: 0.5743, Adjusted R-squared: 0.549
## F-statistic: 22.71 on 6 and 101 DF, p-value: < 2.2e-16

## Stone length - Best model

##
## Call:
## lm(formula = stone length ~ CaCO_3_ + GG + GDD + rainfall + CaCO_3_:GG +
## 1, data = data)
##
## Residuals:
## Min 1Q Median 3Q Max
## -4.0378 -0.8298 0.0001 0.9004 3.5408
##
## Coefficients:
## Estimate Std. Error t value Pr(>|t|)
## (Intercept) 15.2952336 1.3996419 10.928 < 2e-16 ***
## CaCO_3_ 0.0451196 0.0157107 2.872 0.00496 **
## GG1 1.5913964 0.6567381 2.423 0.01715 *
## GDD -0.0008370 0.0002737 -3.058 0.00284 **
## Rainfall 0.0068475 0.0029184 2.346 0.02089 *
## CaCO_3_:GG1 -0.0414480 0.0197916 -2.094 0.03872 *
## ---
## Signif. codes: 0 '***' 0.001 '**' 0.01 '*' 0.05 '.' 0.1 ' ' 1
##
## Residual standard error: 1.414 on 102 degrees of freedom
## Multiple R-squared: 0.2102, Adjusted R-squared: 0.1715
## F-statistic: 5.431 on 5 and 102 DF, p-value: 0.0001803

## Stone width - Best model

##
## Call:
## lm(formula = stone width ~ elevation + CaCO_3_ + GDD + rainfall +
## 1, data = data)
##
## Residuals:
## Min 1Q Median 3Q Max
## -1.60801 -0.26882 0.05209 0.34161 1.25386
##
## Coefficients:
## Estimate Std. Error t value Pr(>|t|)
## (Intercept) 6.0189915 0.5948662 10.118 < 2e-16 ***
## Elevation 0.0005019 0.0003321 1.511 0.133758
## CaCO_3_ 0.0100985 0.0034822 2.900 0.004562 **
## GDD -0.0001754 0.0001175 -1.493 0.138499
## Rainfall 0.0033167 0.0009655 3.435 0.000855 ***
## ---
## Signif. codes: 0 '***' 0.001 '**' 0.01 '*' 0.05 '.' 0.1 ' ' 1
##
## Residual standard error: 0.5062 on 103 degrees of freedom
## Multiple R-squared: 0.2462, Adjusted R-squared: 0.2169
## F-statistic: 8.41 on 4 and 103 DF, p-value: 6.534e-06

## Fruit weight - Best model

##
## Call:
## lm(formula = fruit weight ~ elevation + rainfall + 1, data = data)
##
## Residuals:
## Min 1Q Median 3Q Max
## -1.17938 -0.35419 -0.00816 0.22326 1.79285
##
## Coefficients:
## Estimate Std. Error t value Pr(>|t|)
## (Intercept) 0.6804058 0.4028024 1.689 0.0942 .
## Elevation 0.0005311 0.0002782 1.909 0.0590 .
## Rainfall 0.0023761 0.0009908 2.398 0.0182 *
## ---
## Signif. codes: 0 '***' 0.001 '**' 0.01 '*' 0.05 '.' 0.1 ' ' 1
##
## Residual standard error: 0.5222 on 105 degrees of freedom
## Multiple R-squared: 0.07331, Adjusted R-squared: 0.05566
## F-statistic: 4.153 on 2 and 105 DF, p-value: 0.01837

## Fruit width - Best model

##
## Call:
## lm(formula = fruit width ~ GDD + rainfall + 1, data = data)
##
## Residuals:
## Min 1Q Median 3Q Max
## -4.3165 -0.8804 0.0433 0.7618 4.7618
##
## Coefficients:
## Estimate Std. Error t value Pr(>|t|)
## (Intercept) 11.0888535 1.3317302 8.327 3.35e-13 ***
## GDD -0.0005563 0.0002833 -1.963 0.05224 .
## Rainfall 0.0083538 0.0028116 2.971 0.00368 **
## ---
## Signif. codes: 0 '***' 0.001 '**' 0.01 '*' 0.05 '.' 0.1 ' ' 1
##
## Residual standard error: 1.484 on 105 degrees of freedom
## Multiple R-squared: 0.09814, Adjusted R-squared: 0.08096
## F-statistic: 5.713 on 2 and 105 DF, p-value: 0.004414
